# Geometric Favre-Racouchot syndrome following image-guided superficial radiation therapy

**DOI:** 10.1016/j.jdcr.2025.05.043

**Published:** 2025-07-07

**Authors:** Marshall Hall, Cecilia Nguyen, Henry Lim, Alyssa Forsyth, Wenqin Du, Erica L. Stockbridge, Christian Scheufele, Christopher Wong, Stephen E. Weis, Michael Carletti

**Affiliations:** a Department of Dermatology, Medical City Fort Worth, Fort Worth, Texas; b Division of Dermatology, Department of Internal Medicine and Geriatrics, Texas College of Osteopathic Medicine, UNT Health Fort Worth, Fort Worth, Texas

**Keywords:** Favre-Racouchot syndrome, image-guided superficial radiation therapy, nonmelanoma skin cancer, solar comedone, tobacco

## Introduction

Basal cell carcinoma (BCC) and squamous cell carcinoma (SCC) are the most common types of nonmelanoma skin cancers (NMSCs) diagnosed in Caucasian patients.^1^ The gold standard treatment for NMSC is surgical excision.^1^ However, superficial radiation therapy (SRT) can be used as an alternative treatment.^1^ SRT uses electromagnetic energy generated from X-rays or photons to cause DNA damage.^2^ This leads to cell cycle arrest or apoptosis in rapidly dividing tissues.^2^ Compared to other radiation treatment modalities, SRT deposits energy uniformly and precisely and successful treatment requires lower doses.^2^ Multiple retrospective studies on NMSCs treated with SRT showed comparable cure rates to surgical excision with minor adverse events localized to the treatment site.^2^ Patients and physicians may elect for SRT based on the patient’s comorbid conditions, anatomic location, costs, and those who are surgery averse.^3^^,^^4^ As it is not a surgical procedure, it is said to produce excellent cosmetic results.^3^^,^^4^ Recently, ultrasound image-guidance has been added to SRT to enhance the delivery of SRT.^5^ An ultrasound unit is used to visualize and measure the tumor depth and lateral extent.^2^^,^^5^ This information is used to assess the depth of the lesion to ensure that SRT is an appropriate modality of treatment for the malignancy and assists in determining the optimal amount of energy used during treatment.^5^

Early SRT radiation adverse effects of radiotherapy include alopecia, desquamation, and ulceration.^4^ Late SRT radiation adverse effects include telangiectasia, fibrosis, necrosis, and radiation-induced secondary malignancy.^4^^,^^6^ In addition, there have been a few reported cases of radiation-induced Favre-Racouchot syndrome (FRS) occurring during or after traditional radiotherapy.^7^ The different types of radiation therapy that caused reported radiation-induced acne include deep cobalt therapy, megavoltage therapy, and superficial radiation.^8^ However, to our knowledge, there have not been any reported cases of FRS from NMSC patients undergoing image-guided superficial radiation therapy (IG-SRT). We report 3 patients treated for NMSC with IG-SRT who developed geometric FRS.

## Case reports

### Case 1

**Fig 1:**
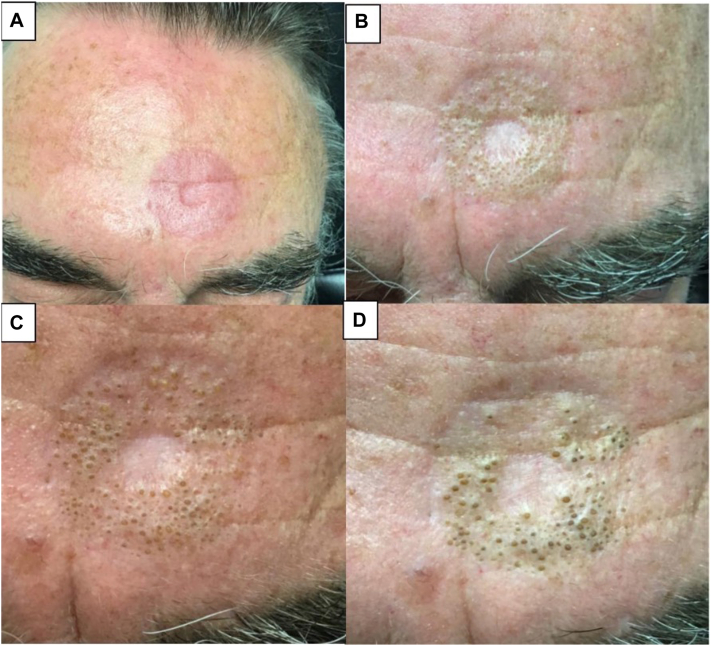
The progression of the comedones during and after IG-SRT. **A,** Shows treatment 10 of 22 (cumulative dose: 2726.20 cGy). **B,** Shows treatment 15 of 22 (cumulative dose: 3089.30 cGy). **C,** Shows 2 weeks post-treatment (cumulative dose: 5667.64 cGy). **D,** Shows 3 months post-treatment. *IG-SRT*, Image-guided superficial radiation therapy.

A 68-year-old male who was receiving IG-SRT for a nodular BCC on the forehead and right arm presented with a new lesion on his forehead (Fig 1). He initially noticed the lesion on his forehead within the radiation treatment field during the sixth week of treatment (fraction number: 15, cumulative dose: 4089.30 cGy), about 75% of the way through his treatments. He denied any lesions arising from the radiation treatment field on his right arm. He also has a history of coronary artery disease, peripheral neuropathy, and tobacco use (<1 pack per day).

Physical examination showed a well-demarcated, round geometric plaque on the central forehead with numerous open comedones within the radiation field. At the central aspect of the treatment site, where the diagnostic biopsy was taken, there was a loss of adnexal structures without comedone formation. The radiation site of the right arm showed no comedone formation. The patient completed his IG-SRT to each of these lesions without additional adverse events (fraction number: 22, cumulative dose 5667.64 cGy).

The patient was treated with adapalene 0.1% gel nightly. Six months after completing IG-SRT, he was treated for another nodular BCC on the right clavicle with IG-SRT. No comedones formed at the clavicular or the previously treated forearm treatment sites. The lesion on the forehead remained within the treatment area. Minimal to mild improvement was noted during 7 months of observation. He was lost to follow-up after the completion of his second course of IG-SRT.

### Case 2

**Fig 2:**
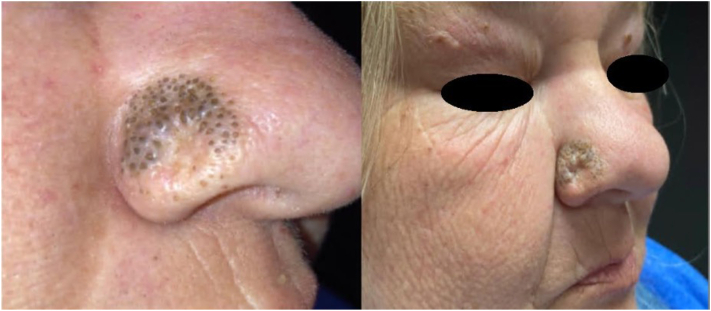
Treatment site 3 months following treatment with IG-SRT. There is a well-demarcated geometric plaque of open comedones restricted to the radiation field with a central scar at the location of the treated nodular BCC. *BCC*, Basal cell carcinoma; *IG-SRT*, image-guided superficial radiation therapy.

A 59-year-old female with a history of nodular BCC on the right nasal ala, which was treated with IG-SRT, complained of a lesion at the treatment site on her nose (Fig 2). The lesion was first noted during her 2-week follow-up visit after completing her radiation treatments (fraction number: 22, cumulative dose: 6124.8 cGy). She reported scaling, irritation, and burning to the affected area. Additional history included hypertension, dyslipidemia, migraines, coronary artery disease, and tobacco use (1/2 pack per day).

Physical examination showed a well-demarcated, geometric round plaque with numerous open comedones at the IG-SRT treatment site on the right nasal ala. There was a central scar with no adnexal structures and no comedones at the malignancy diagnostic biopsy site. At this time, the patient was recommended to apply adapalene 0.1% gel nightly to the affected area.

**Fig 3:**
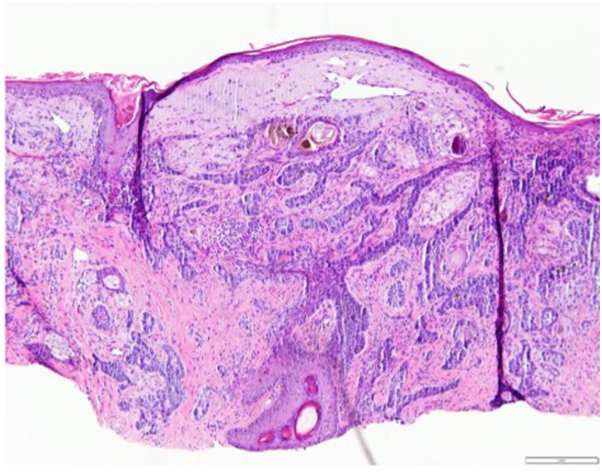
Histopathology shows dilated follicular orifices filled with cornified debris and slight inflammation (Hematoxylin-eosin stain, 200× magnification).

At her 3-month and 6-month scheduled follow-up, there was increased prominence of the lesion isolated to the area of radiation. At the 6-month follow-up, a biopsy and curettage of the lesion was performed. Histology showed several dilated follicular orifices filled with cornified debris forming multiple comedones (Fig 3).

At follow-up, it was noted that the majority of the comedones had not recurred and remained stable approximately 1 year after performing the biopsy and curettage. A few open comedones remained. The patient was not cosmetically or symptomatically concerned about the remaining lesions and declined further treatment.

### Case 3

**Fig 4:**
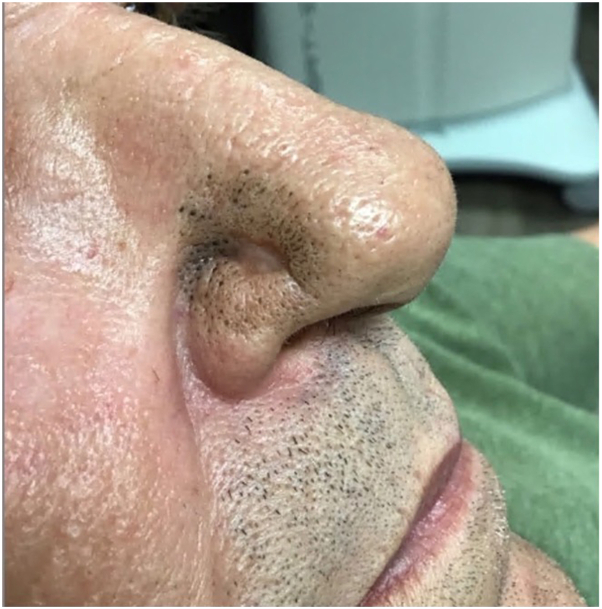
On the right nasal ala is a geometric well-demarcated round plaque with open comedones throughout the plaque with a central scar at the site of treated BCC. *BCC*, Basal cell carcinoma.

A 61-year-old with a nodular BCC on the right nasal ala was treated with IG-SRT. 3 weeks after completing treatment (fraction number 20, cumulative dose 5579.2 Gy), he presented for his post-treatment follow-up visit with a lesion on his nose (Fig 4). The lesion was asymptomatic. His medical history includes systolic cardiomyopathy, atrial fibrillation, coronary artery disease status post coronary artery bypass graft, hypertension, hyperlipidemia, cannabis use, amphetamine, and tobacco use (1-2 packs per day). Physical examination showed a well-demarcated round plaque with open comedones throughout the plaque with a central scar at the diagnostic biopsy site, similar to the cases above. The patient was recommended to use adapalene 0.1% gel to the affected area.

## Discussion

FRS, also known as solar comedones, senile comedones, and nodular elastosis, is a common cutaneous disease that is clinically characterized by multiple open and closed comedones and cysts in actinically damaged skin.^7^^,^^9^ Additionally, the skin may take on a yellow hue and may be atrophic.^10^ It is commonly distributed in chronically sun-exposed areas of the face, such as the bilateral periorbital and temporal skin.^7^^,^^9^ FRS primarily affects men with a light complexion between the ages of 40 and 60 years.^9^ An estimated 1.4% of the adult population aged between 25 and 74 years had FRS and up to 6% of Caucasian adults aged more than 50 years.^11^ It has an indolent course, and documented associated risk factors include excessive chronic ultraviolet (UV) radiation exposure, heavy cigarette smoking, and radiation therapy.^7^^,^^9^

The first reported case of radiation-induced acne was by Bluefarb in 1947.^12^ Since then, there have been reported cases of radiation-induced FRS occurring during treatment, months to years after radiation therapy.^7^^,^^8^ Prior reported cases include patients being treated with radiation therapy for oligodendroglioma, astrocytoma, SCC, BCC, and melanoma.^8^^,^^11^, ^12^, ^13^, ^14^ Radiation modalities that have been reported to cause FRS include orthovoltage, megavoltage, and cobalt radiation.^12^ Some of the patients had additional treatments, such as surgery or chemotherapy, before or after radiation therapy.^8^^,^^14^ The patients with reported FRS were located on the face or neck and occurred shortly after completion of treatment.^8^^,^^12^, ^13^, ^14^ Similar to our patients, the lesions were localized to the previously irradiated area. Additionally, some of the patients had a history of tobacco use. All of the patients in our cases were actively smoking tobacco products throughout their radiation treatments. Our cases differ from the prior reported cases due to the form of radiotherapy being used and the geometric pattern of FRS.

It is important to note that the patient in Case 1 had additional NMSCs on the forearm and trunk. The forearm BCC was treated at the same time as the forehead lesion. The trunk lesion began treatment 6 months after the forehead and forearm lesions were treated. Neither of these other treatment sites developed comedones. This suggests that the location of the lesion may play a role in the development of FRS, especially in those actively smoking tobacco products during treatment. All 3 patients who developed FRS were treated for nodular BCC. It is unknown if there is a high predisposition for patients with this type of malignancy to develop FRS. It is possible that because BCC is the most common cutaneous malignancy, FRS is more likely to be seen with this malignancy. It is unknown if the underlying pathophysiological changes from BCC are more likely to cause FRS compared to SCC.^15^

While the precise pathogenesis of FRS is not well understood, it is likely due to a combination of UV skin damage, acnegenic drugs, radiation, and tobacco exposure.^7^^,^^9^^,^^12^ It is believed that chronic UV exposure damages the elastic network in the upper and mid dermis through oxidative stress, causing dermal and epidermal atrophy and disorganized and thickened elastic fibers.^9^^,^^14^ As a result, these changes facilitate sebum retention and keratinization of pilosebaceous follicles.^14^ This creates an environment conducive to comedone, nodule, and cyst formation.^14^ Drugs that can cause acne include steroids, isoniazid, anticonvulsants, and luteinizing hormone-releasing hormone analogs.^12^ More specifically, androgens can cause comedogenesis by activating androgen receptors located on cells of pilosebaceous ducts.^12^

Radiation therapy and tobacco use can cause degenerative changes in connective tissue stroma similar to solar elastosis.^9^^,^^10^ We hypothesize that the degenerative changes caused by tobacco work synergistically with underlying actinic damage and radiation exposure, increasing the risk of developing FRS. Additionally, the high concentration of sebaceous glands on the face may also contribute to its formation. This theory is supported in Case 1 where the patient developed comedones after IG-SRT to the lesion on his forehead, but not at the treatment sites on his forearm and trunk, which are areas less exposed to tobacco smoke. In these cases, a combination of sun-damaged skin, radiation treatment, and direct contact with tobacco smoke may be the mechanism by which these comedones form.

With these outcomes in mind, it is important to monitor for adverse events and complications of IG-SRT. IG-SRT for some patients can be the ideal treatment of NMSC tumors with early staging.^5^ It is well tolerated and has excellent tumor control using ultrasound to adjust radiation energy and dose in comparison to SRT without ultrasound imaging.^5^ One of the reasons patients may choose treatment with IG-SRT is because of its cosmetic outcomes. The tissue-sparing property of IG-SRT makes it ideal for treating anatomically and cosmetically sensitive and high-risk regions, such as the head and neck.^5^ However, as is seen in these cases, IG-SRT does not always produce optimal cosmetic results. While this adverse event is rare, it is important to include this in discussions when deciding on IG-SRT and show the sequelae of SRT to demonstrate that while this procedure may produce good cosmetic results for some patients, there are also cases with poor cosmesis. Additionally, discussions regarding tobacco use while undergoing IG-SRT may be beneficial to prevent FRS. Further studies are needed to understand the correlation between IG-SRT, solar elastosis, tobacco use, and FRS. When patients are being treated with IG-SRT on the face, we recommend patients discontinue tobacco products prior to beginning IG-SRT up until they have completed their radiation sessions and the skin has healed.

## Conclusion

To our knowledge, these are the first 3 reported cases of FRS secondary to IG-SRT. As IG-SRT becomes increasingly more prevalent as a treatment for NMSC, it is important to be cognizant and monitor for potential adverse events and complications. Solar comedones at the site of radiation therapy, as seen in our presented cases, may be a deterrent for patients to select this as a treatment. Given these complications, it is important to discuss that while it may produce good cosmetic outcomes, poor cosmesis may still occur. Thus, further investigation is needed to assess ways to mitigate these risks while providing optimal patient care. Finally, when patients are treated with IG-SRT on the face, we recommend discontinuation of tobacco products until radiation treatment sessions have been completed and the skin has healed.

## Conflicts of interest

None disclosed.
